# Effects of Arsenic-Eating

**Published:** 1876-08

**Authors:** 


Effects of Arsenic-Eating.—A late observer in Styria, Dr. Knappe, says:

Arsenic-eaters are, in all cases with which I am acquainted, of healthy appearance, and with sexual desires strongly developed. I am of opinion that only strong persons can accustom themselves to it. They attain sometimes great age. I saw in Zeiring a still very strong charcoal-burner at the age of 70, who had taken arsenic, it was believed, for forty years. I have never noticed in habitual arsenic-eaters any arsenical cachexia. It happened, however, that an arsenic-eater, a currier's apprentice in Liegist, 1865, took while intoxicated an over-dose, and brought upon himself an acute attack of poisoning. According to his statement, he had taken a piece the size of a bean. He recovered, however, and again took to arsenic-eating, but with more circumspection.—*Med. and Surg. Reporter.*

